# Development and validation of a pharmacist-led education model in allergic rhinitis management: a multi-phase study

**DOI:** 10.1186/s40545-023-00625-1

**Published:** 2023-10-04

**Authors:** Chii-Chii Chew, Xin-Jie Lim, Pathma Letchumanan, Maithrea Suresh Narayanan, Philip Rajan, Chee Ping Chong

**Affiliations:** 1 Discipline of Clinical Pharmacy, School of Pharmaceutical Sciences, Universiti Sains Malaysia, 11800 Minden, Penang Malaysia; 2 Clinical Research Centre, Hospital Raja Permaisuri Bainun, Ministry of Health, Level 4, Ambulatory Care Centre (ACC), Jalan Raja Ashman Shah, 30450 Ipoh, Perak Malaysia; 3 Department of Otorhinolaryngology—Head and Neck Surgery, Hospital Raja Permaisuri Bainun, Ministry of Health, Ipoh, Perak Malaysia

**Keywords:** Rhinitis, Allergic, Pharmacist, Education, Pharmaceutical services

## Abstract

**Background:**

Patient education is identified as one of the core and fundamental management strategies in the management of allergic rhinitis. The Allergic Rhinitis and its Impact on Asthma (ARIA) guidelines developed guidance for the management of allergic respiratory disease, and the guidelines are applicable to the international context. The ARIA guidelines for the pharmacy have specifically encouraged the creation of local pharmacist-led intervention in allergic rhinitis management. This study aims to develop a pharmacist-led educational model using a multi-phase study approach.

**Method:**

In phase one, we conducted a literature review using four databases to extract relevant articles and clinical practice guidelines published between 2017 and 2022. The information was structured into a questionnaire consisting of patient education material (10 domains with 130 items) and pharmacist counseling scopes (15 domains with 43 items), with each item having a rating scale ranging from 1 (lowest) to 9 (highest) level of agreement. Fifty-two panellists, including otorhinolaryngologists and pharmacists, were invited to complete the questionnaire. A consensus agreement was considered when at least 70% of panellists scored 7 to 9 (critically important). A two-round survey was conducted, and descriptive analysis, inter-rater reliability (≥ 0.5–1 indicate moderate to excellent reliability), variation in the relative interquartile (VRIR < 0.3 indicate good stability), and variation in the coefficient of variation (VCV < 40% considered consensus achieved) were performed. In phase two, patient education material was developed into audio-visual format, and in phase three, patients rated its understandability and actionability using a validated Patient Education Materials Assessment Tool.

**Results:**

In the round one Delphi survey, 43 panellists responded, with 171 out of 173 items achieving “consensus agreement” (75.4–100%). In the second survey, 32 out of 43 panellists responded, with most items (171 out of 173 items) stable across rounds and all items had acceptable internal consistency (VCV: − 12.21–15.81). Two items did not achieve “consensus agreement” (64%) but improved in round two (92.9%), however, instability was observed (VRIR: 0.36). These two items were retained in the model due to achieving the minimum level of agreement and internal consistency (VCV = 15.81). Inter-rater reliability was 0.608 and 0.970 in the respective rounds. Patients rated the educational material as understandable (81.8–100%) and actionable (100%).

**Conclusion:**

The validated pharmacist-led education model, with its educational materials tested on end-users, provides structured patient education and pharmaceutical care in assisting patients with allergic rhinitis. The educational material allows the delivery of standardized information by the healthcare providers to the patients. Further research on the effectiveness of this model in improving patients’ symptom control and quality of life is warranted.

**Supplementary Information:**

The online version contains supplementary material available at 10.1186/s40545-023-00625-1.

## Introduction

Allergic rhinitis (AR) is an inflammatory reaction following allergens exposure, causing symptoms like rhinorrhoea, nasal congestion, and itchiness [1]. It results in significant financial burdens on healthcare, with annual costs in the U.S.A. reaching 3.4 billion USD while indirect cost accounted for 5.2 billion USD in 1996 [2]. The increasing prevalence of AR has led to a global prevalence of 1.0–54.5% up to 2020 [3]. The healthcare cost and economic burden are primarily due to the increasing prevalence of AR in industrialized countries [4].

AR is a chronic condition requiring patients’ self-management and healthcare providers’ support [5]. The fundamental of management involves educating patients on understanding disease nature, allergens avoidance, and medication adherence [1, 6–8]. However, there is a significant knowledge gap, with a range of 22–70.1% of patients having knowledge about AR [9, 10]. Non-adherence to intranasal corticosteroid is prevalent among AR patients (36.9–67.3%) [11–13]. Patients’ lack of knowledge and skill was predictor of non-adherence [6]. The current educational material online is not standardized, the techniques of intranasal spray administration have differed across online information [12, 18–21], leading to conflicting information for patients.

Pharmacist-led educational interventions improve patients' quality of life and symptom severity [14]. Pharmacists significantly influence patients' management of AR [15, 16] by setting goals for care and providing pharmacist-led educational interventions [17, 18]. The roles of pharmacists are included in the integrated pathway (ICPs) of Allergic Rhinitis and its Impact on Asthma (ARIA) guidelines [19], which encourage the development of local standard pharmaceutical care guides for AR management [20]. ARIA is a non-governmental organization that educates, advocates and puts evidence-based management of AR and asthma into practice worldwide. The first ARIA documents were made by a panel of experts in 1999 that provide a basis for healthcare providers and health organizations to develop country-specific local standards of care [21].

This study aimed to develop and validate a pharmacist-led education model through a multi-phase approach, considering patient education and the need for local pharmaceutical care guidance.

## Methods

**Fig. 1:**
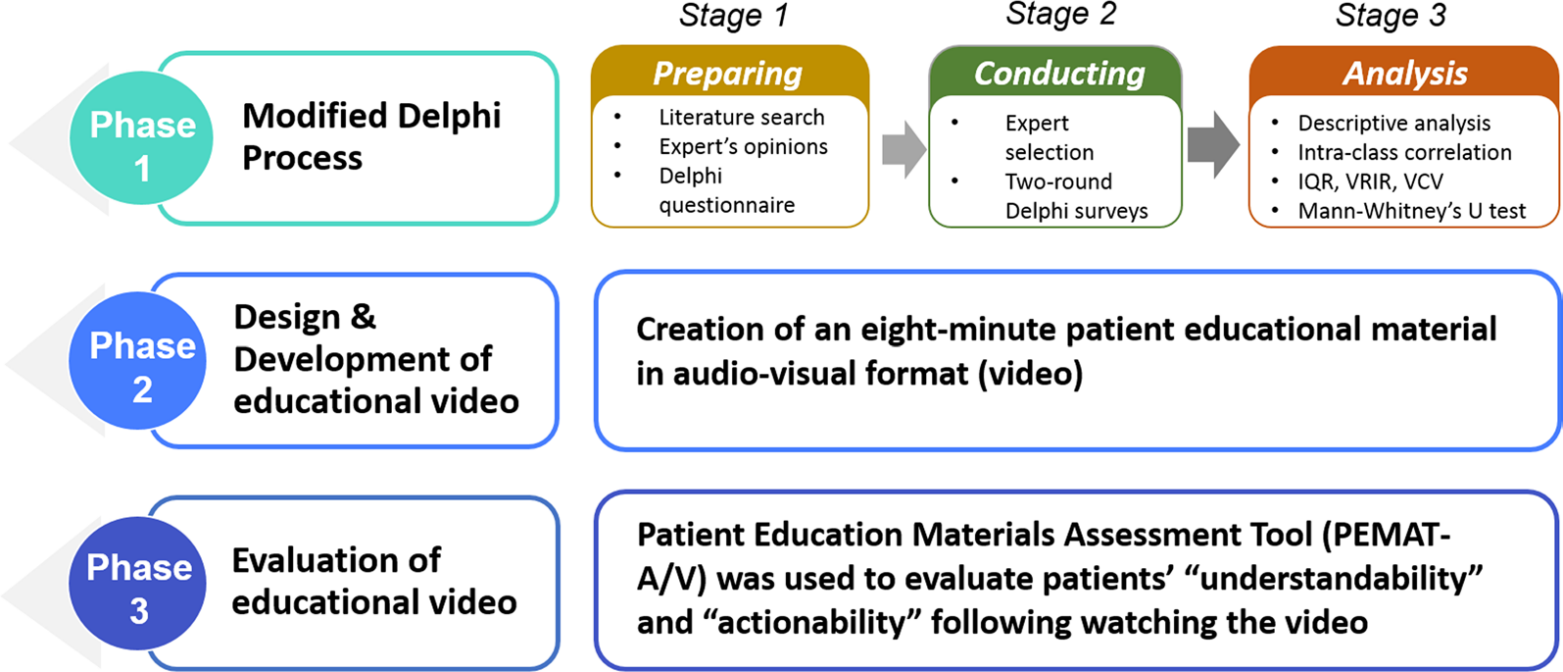
An overview of developing pharmacist-led education material in multi-phases

This study aimed to present a valid and reliable pharmacist-led education model for educating AR patients. This material was developed using a methodological approach in three phases: (1) the modified Delphi process; (2) the design and development of the educational video; (3) the evaluation of the educational video (Fig. 1).

### Phase 1: modified Delphi process

The Delphi technique is a systematic method for controlling group communication to address an issue as a whole [22]. It has increasingly been used in pharmacy practice research to achieve consensus on knowledge, roles, competencies, medication literacy service optimization, and a collaborative model [23].

We adopted the modified Delphi method that used structured items with a 9-point Likert scale in round one Delphi survey. This quantitative technique was used due to readily available research model development information [23, 24]. This technique helped to simplify consensus-building in respective domains [25]. The two-round modified Delphi process utilized controlled feedback on expert input to obtain consensus among a panel of experts through three stages: preparing, conducting, and analysing [26] (Fig. 1).

#### Preparing

The Delphi process was started on a literature review of academic journals, randomised controlled trials, international and local clinical practice guidelines, and reviewed articles. A search for practice guidelines and journals in the management of AR was performed on different databases, including PubMed, ScienceDirect, SpringerLink, and EBSCOHost. The keywords entered included “allergy rhinitis and practise guidelines”, “clinical practice guidelines”, “patient education”, “pharmacist-led educational protocol”, “pharmacist-led intervention”, and “AR”.

The relevant literature published between 2017 and 2022 in the English language by excluding articles with the title of “paediatric”, “child”, “children”, “infant”, and “adolescent” were searched. Practice guidelines from various countries, including the United States, United Kingdom, Canada, and Malaysia, were compared to the Allergic Rhinitis and its Impact on Asthma (ARIA) guideline. The initial search generated over 604 articles gathered in the Zotero citation manager to identify duplication. The principal investigator, who has research experience in otorhinolaryngology, read these articles and removed irrelevant ones. Finally, 37 articles were reviewed for potential competencies. A list of 173 competencies items was generated, with the definition, pathology, and causes of AR were adapted from the first ARIA guidelines developed in year 2008 [21].

A superior reference panel, including consultants, an otorhinolaryngology specialist, and researchers, aimed to construct, define, and set domains for the study model. A consensus meeting was held to confirm competency items [27]. Senior pharmacists discussed competencies items related to pharmacy practice. The Delphi questionnaire, including patient education information and pharmacist counselling scope, was finalized by an academic supervisor. A nine-point Likert scale from very low (1) to very high (9) agreement levels was used. An open response field for additional comments was added to gain extra insight. The domains and the associated items are presented in Table 1.

**Table 1:** The domains and number of items of the Delphi survey questionnaire

Domains	Number of items
l. Patient education information	
Background on the disease	4
Symptoms	5
Diagnosis	5
Allergen identification and avoidance	19
Information on corticosteroid nasal sprays	15
How to use them	19
The tips of effective administration	5
Nasal spray cleaning	6
Antihistamines	10
Decongestants	19
Nasal saline	13
What to do when symptoms get worse	6
What happens when AR is not well controlled	4
II. Pharmacist counselling scopes and algorithm	
Patient selection criteria	1
Symptom control assessment and monitoring	1
Quality of life assessment	1
Setting the goal of treatment	1
Demonstrating the technique of intranasal corticosteroid administration	2
Assessing the technique of using nasal spray device	2
Providing guidance in assessing concerns of using nasal spray	5
Teaching the patient an alert sign	3
Conducting follow-up pharmaceutical care when necessary	1
Patient discharge criteria	1
Pharmaceutical care algorithm in the management of AR	1
Listed guidance in addressing patients’ concerns about treatments	6
Discussed with patients about medication adherence	4
Adapted ARIA guideline stepwise treatment approach	1
Pharmacotherapy agents of AR treatments	13

#### Conducting

I. Expert panel
  The criteria for expert panel selection include (i) otorhinolaryngologist; (ii) family medicine specialist; (iii) medical doctor with at least 5 years of experience in the Otolaryngology department; (iv) pharmacist with five years of experience in the hospital pharmacy practices [28]. The literature suggests that the minimum number of panellists ranges from 10 to 18 per area of expertise [29]. The study team sampled a minimum of 10 panellists from each area of expertise. Based on a similar study, a ratio of medical doctors to pharmacists 3:1 was used to sample the panellists [30], resulting in a sample size of 30 medical doctors and 10 pharmacists. By estimating 20% of the non-response rate, the research team invited 37 medical doctors and 15 pharmacists. A minimum response rate of 70% is required for a Delphi survey round (65).
II. Delphi survey rounds
  *Round one* In round one (R1), the panellists were invited via telephone calls, email, and face-to-face meeting. The questionnaires were sent to them by email (*n* = 32) and hand-delivered (*n* = 20). Each panellist was given a 2-week period to complete the first round of the survey. Those who were yet submitted their comments after the due date were given a 1-week extension and an email to remind them [31]. After the first round, the data were analysed to determine the individual and group median scores.
  *Round two* In round two (R2) of the Delphi survey, the questionnaire was sent to them by email (*n* = 20) and hand-delivered (*n* = 23). Each panellist was sent a first-round report illustrating each item’s individual and group median scores. Participants were invited to re-rate the consensus-building process only for items needing clarification, not achieving a “consensus agreement”, or new items suggested by the panellist in R1 via free-text responses. Up to two email reminders were sent to request completion [31].
  *Analysis* The data analysis was performed using SPSS version 20.0 and Microsoft Excel^®^. The consensus of the agreement (%) for each item was calculated as the number of respondents rated at a particular score divided by the total of panel responses to that item. The central tendency and dispersion measures were calculated [24, 32, 33].

In the first scenario, a “consensus agreement” was defined as 70% of the panellists scoring 7–9 [34]. The score within this range indicated a “critically important” item and would be accepted and acknowledged in the subsequent round without having to re-rate [32, 33, 35]. Besides, the consensus for an item is considered achieved by using an alternative criterion of the interquartile range (IQR) ≤ 2.00 on a 9-point scale [36].

In the second scenario, a “consensus disagreement” is defined as 70% of the panellist scoring 1–3, where these scores are defined as “not important item”, and would not be presented to the next round. They would be dropped from the model [32, 33, 35]. No specific cut-off of IQR value indicates absolute disagreement. An IQR of 3.00 signify the least agreement was adapted as a reference point [37].

In the third scenario, the items which scored 4 to 6 are considered “important but not critical” to include in the model and would be brought forward to the next round for re-rating [32, 33, 35].

In the fourth scenario, the items that received comments from the panellist were collated for discussion among the study team members. Literature reviews were performed to confirm the panellists’ suggestions [24, 32]. The items with changes made were presented in the round two surveys for re-rating.

Triangulation strategies were used to determine consensus in Delphi survey rounds, using parameters like IQR, variation in the relative interquartile (VRIR), and variation in the coefficient of variation (VCV). Mann–Whitney’s *U* test analysis was used to check statistically significant differences between rounds, with a *p*-value less than 0.05 [33].

The study assesses proximity responses among panellists to achieve consensus, using an IQR of less than 0.5. Group stability is achieved when the relative interquartile range (VRIR) variation is less than 0.30. Internal agreement is indicated by a coefficient of variation (VCV) of less than 40% between rounds [33]. Inter-rater reliability is determined using intra-class correlation (ICC) estimates. The ICC 95% confidence intervals were calculated using a mean rating (k = 43), absolute agreement, and a 2-way mixed-effects model [38].

### Phase 2: design and development of the educational video

The research team created an 8-min educational video using the Canva^Ⓒ^ software programme. Each statement was supported by a colourful illustration and audio description that was easily understood. The topics covered in the video include AR definition, causes, risk factors, disease nature, symptoms, and methods to effectively manage the symptoms. The methods involved allergen identification and avoidance strategies, the importance of regular use of intranasal corticosteroids, and attending follow-up at the clinic. The measures to be taken when symptoms become worse, and the consequences of inadequate control of AR were included in the last part of the video.

### Phase 3: evaluation of the educational video

A Patient Education Materials Assessment Tool (PEMAT-A/V) [39] was used to evaluate audio-visual patient education materials. The PEMAT-A/V tool (English) was validated by the Agency for Healthcare Research and Quality [40]. This tool had a moderate agreement per Kappa (average K = 0.57), strong agreement per Gwet’s AC1 (average = 0.74), and strong internal consistency (α = 0.71; average item-total correlation = 0.62). The tool evaluates end-user’s “understandability” and “actionability” in patient education materials. “Understandability” domain assesses content, word choice, style, organization, layout, design, and visual aids. The “Actionability” domain evaluates their ability to take action. The domain of “Understandability” consists of 11 questions after excluding two questions that did not apply to video format, while “Actionability” has 4 questions [40].

A Malay-translated PEMAT-A/V (M) version with excellent inter-rater reliability 0.791 (95% CI: 0.635–0.915) and 0.733 (95% CI: 0.559–0.887) in understandability and actionability domains, respectively, was used in this study. PEMAT-A/V (M) was adapted for the local population and obtained permission from the originator [39].

The PEMAT-A/V uses a scoring system (scale of 0 to 100%) to generate the score in percentage by summing the score of each item, whereby 1 point is given to the item marked “agree” and 0 points to disagree. The total score was divided by the total number of items and multiplied by 100% to generate each score for “understandability” and “actionability”. A cut-off of 70% is used to determine whether the patient education material in the form of video is sufficiently “understandable” and “actionable” by the end-users [40]. Seventy-nine patients with AR were estimated using a G* power sample size, with an effect size of 0.2 based on Cohen's d [41], a constant proportion of 0.5, a significance level of 0.05, and a power of 0.95. A consecutive sampling technique was employed to recruit patients who diagnosed with AR attending the outpatient otorhinolaryngology clinic.

## Results

### Expert panel

In R1, of the 52 panellists invited, 25 otorhinolaryngologists, 13 pharmacists, 4 senior medical doctors, and a family medicine specialist responded (response rate: 82.7%). They had working experience of 6–27 years, and the estimated proportion of AR patients managed in a month ranged from 25 to 75% (Table 2). The R2 received a response rate of 74.4%, with 32 out of 43 panellists responding.

**Table 2:** Demography details of the panellist

Variables		*n* = 43; *n* (%)
Age, mean (SD)		42.1 (5.8)
Mean work experience, mean year (SD)		16.6 (4.7)
Gender		
Male		20 (46.5)
Female		23 (53.5)
Profession		
Otorhinolaryngologist		22 (51.1)
Otorhinolaryngologist in academia		4 (9.3)
Senior Medical Doctor		3 (7.0)
Pharmacist		11 (25.6)
Pharmacist in academia		2 (4.7)
Family medicine specialist		1 (2.3)
Work institution		
Government-funded hospital		32 (74.1)
Government-funded clinic		3 (7.0)
University hospital		4 (9.3)
Others		
Government health office		1 (2.3)
State health department		1 (2.3)
University		2 (4.6)
The estimate proportion of patients with AR (AR) managed in a month		
None		4 (10.0)
25%		17 (42.5)
50%		10 (25.0)
75%		8 (20.0)
100%		1 (2.5)
Missing		3

### Inter-rater reliability

In the R1, the reliability among panellists was moderate with an ICC estimate of 0.608 (95% CI 0.512–0.695), but improved to excellent reliability with an ICC estimate of 0.970 (95% CI 0.945–0.987) in the R2 survey.

### Two-round Delphi survey

#### Patient educational material

**Fig. 2:**
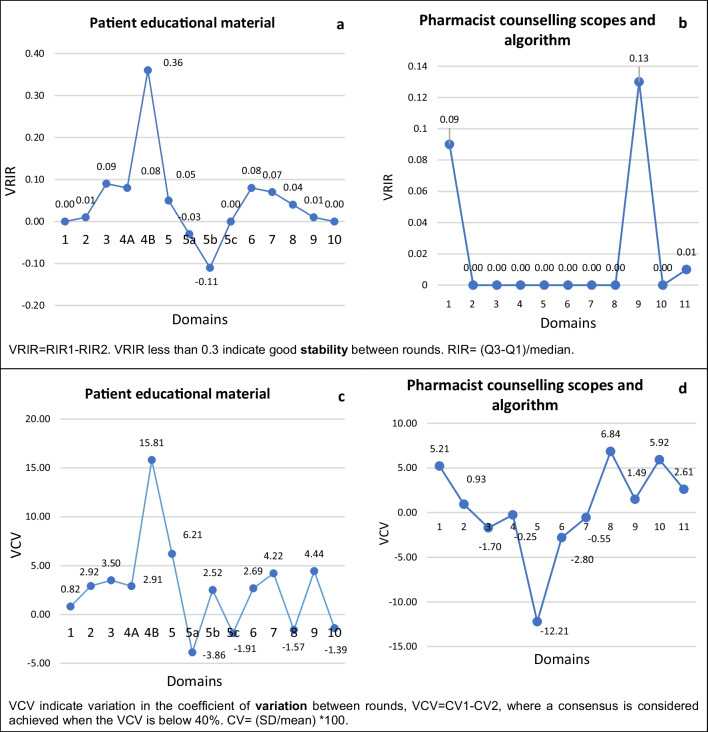
**a** and **b** Indicate variation in the relative interquartile range (VRIR) between rounds, while **c** and **d** represent variation in the coefficient of variation (VCV)

In R1, a “consensus agreement” was achieved for 9 domains consisting of 111 items, with 85.8 to 95.9% rated critically important. However, two out of 19 items under the domain “allergen identification and avoidance” did not meet “consensus agreement”, with 64.0% of the panellists rating them as critically important. These items had a median score of 7.50 (IQR: 5.00–9.00), indicating the least agreement among the panellists. The statements of these two items were “wearing goggles” and “keeping doors and windows closed”. Their comments were that “what types of goggles” and "the instruction of execution were not explicit", respectively. Panellists suggested that “types and timing of wearing goggles” and "the timing for patients to take action” be specified (Additional file 1: Appendix I: Rating of panellist in R1 survey). The panellist's suggestions were adopted and brought the items forward for re-rating in subsequent rounds. In R2, the median score improved to 8.50, with 92.9% rating the items as critically important (Table 3). Although there was slight group instability between rounds (VRIR = 0.36) (Fig. 2a), the VCV was 15.81% (< 40.0%), indicating a consensus was achieved.

**Table 3:** Comparison of R1 and R2 ratings

Domain	No. of item^	R1						R2						*z*	*p*-value
		Mean	SD	Median score	Q1	Q3	Critically important	Mean	SD	Median score	Q1	Q3	Critically important		
Patient educational material															
1. Knowledge of the disease	1	8.42	0.98	9.00	8.00	9.00	97.7%	8.41	0.91	9.00	8.00	9.00	97.0%	0.000	1.000
2. Symptoms assessment	3	8.14	1.13	8.33	7.50	9.00	90.8%	8.30	0.91	8.75	7.75	9.00	95.5%	− 1.000	0.317
3. Diagnosis	1	7.42	1.68	8.00	6.50	9.00	75.0%	7.94	1.52	8.00	7.25	9.00	91.4%	0.000	1.000
4. A) Allergen identification and avoidance	9	7.87	1.39	8.33	7.11	9.00	86.4%	8.20	1.21	8.80	7.80	9.00	93.8%	− 1.000	0.317
B) Two items of allergen identification and avoidance^§^	2	6.90	2.01	7.50	5.00	9.00	64.8%	8.18	1.09	8.50	7.50	9.00	92.9%	− 1.000	0.317
5. Nasal corticosteroid	6	7.95	1.45	8.33	7.33	9.00	89.4%	8.23	0.99	8.71	7.71	9.00	92.2%	− 1.000	0.317
a. Technique of priming	4	8.48	0.89	9.00	8.13	9.00	92.9%	8.22	1.18	9.00	7.88	9.00	90.9%	0.000	1.000
b. Before administration	1	8.16	1.76	9.00	8.00	9.00	90.9%	8.03	1.53	9.00	7.00	9.00	91.2%	0.000	1.000
c. During administration	1	8.42	0.99	9.00	8.00	9.00	93.2%	8.27	1.13	9.00	8.00	9.00	94.1%	0.000	1.000
6. Antihistamine	3	7.98	1.41	8.67	7.33	9.00	81.8%	8.21	1.23	9.00	8.00	9.00	90.3%	− 1.000	0.317
7. Decongestant	2	7.79	1.72	8.50	7.00	9.00	83.0%	8.23	1.47	9.00	8.00	9.00	95.6%	− 1.000	0.317
8. Nasal douche (saline)	5	8.03	1.22	8.30	7.10	9.00	87.3%	8.23	1.38	9.00	8.00	9.00	93.3%	− 1.000	0.317
9. What to do when symptom flare	3	8.18	1.21	8.83	7.50	9.00	90.2%	8.31	0.86	8.50	8.00	9.00	96.0%	− 1.000	0.317
10. Consequences of non-adherence	2	8.27	1.00	9.00	8.00	9.00	92.1%	8.31	1.12	9.00	8.00	9.00	93.8%	0.000	1.000
Pharmacist counselling scopes and algorithm															
1. Patient selection criteria	1	7.88	1.37	8.00	7.25	9.00	88.1%	8.13	0.99	8.00	8.00	9.00	90.6%	0.000	1.000
2. Symptoms assessment	1	8.00	1.29	8.00	7.00	9.00	88.4%	8.03	1.22	8.00	7.50	9.00	93.8%	0.000	1.000
3. Patients’ quality of life	1	8.23	1.01	9.00	8.00	9.00	87.5%	8.23	1.15	9.00	8.00	9.00	87.5%	0.000	1.000
4. New user to nasal spray	1	8.57	0.70	9.00	8.00	9.00	100.0%	8.55	0.72	9.00	8.00	9.00	96.9%	0.000	1.000
5. Existing nasal spray users	1	8.55	0.71	9.00	8.00	9.00	97.7%	8.19	1.68	9.00	8.00	9.00	90.6%	0.000	1.000
6. Teach patients the alert sign	1	8.55	0.77	9.00	8.00	9.00	97.7%	8.47	1.00	9.00	8.00	9.00	100.0%	0.000	1.000
7. Follow-up pharmaceutical care	1	8.26	1.17	9.00	8.00	9.00	90.7%	8.29	1.22	9.00	8.00	9.00	96.9%	0.000	1.000
8. Patient discharge	1	8.23	1.16	9.00	7.00	9.00	93.0%	8.55	0.62	9.00	8.00	9.00	100.0%	0.000	1.000
9. Algorithm of pharmaceutical care	1	8.13	1.03	8.00	8.00	9.00	92.3%	8.50	0.95	9.00	8.00	9.00	96.3%	− 1.000	0.317
10. Stepwise treatment approach	1	8.00	1.55	9.00	8.00	9.00	86.5%	8.40	1.13	9.00	8.00	9.00	93.5%	0.000	1.000
11. Pharmacotherapy agents	3	8.28	0.99	8.88	8.00	9.00	92.8%	8.45	0.79	9.00	8.00	9.00	98.0%	− 1.000	0.317

^The number of items re-rated for the round two survey

Q1 and Q3 indicate quartile one and three, respectively. *Z* scores and *p*-value were analysed using Mann–Whitney’s U test

^§^The statements of these two items were improvised

In R2, 90.9–97.0% of panellists rated all items as critically important. The relative interquartile range between rounds showed group stability (VRIR = − 0.11–0.36), with variation within acceptable ranges (− 3.86 to 15.81%). Mann–Whitney’s *U* test did not show significant differences in ratings between rounds at all domains (*p* = 0.317–1.000, *z* = − 1.000 to 0.000) (Table 3).

#### Pharmacist counselling scopes and algorithm

The R1 found that 15 domains with 43 items achieved consensus agreement, with 86.5–100% rating them as critically important. In R2, 87.5–100% of panellists rating the items as critically important (Additional file 1: Appendix I). Between-round variations were stable across 0.00 to 0.13 (VRIR) and − 12.21 to 6.84% (VCV) (Fig. 2b and d).

The first and ninth domains showed slightly higher VRIR, 0.09 and 0.13, respectively (Fig. 2b). The first domain focused on patient selection criteria with the inclusion of moderate-to-severe AR for counselling and management, while the ninth domain involved a flow chart for stepwise patient assessment and counselling. The panellists suggested that patients with all levels of severity should be included, and the flowchart structure was improved for better clarity. Improvements were observed in the mean score, IQR, and percentage of consensus agreement in R2 (Table 3).

The highest variation between rounds was observed in the "Evaluation of existing nasal spray users" (VCV = − 12.21). The R1 statement was to evaluate patients' corticosteroid nasal spray administration technique and correct it when necessary. However, some panellists deemed it unnecessary due to the heavy patient load. A statement “considering patients with uncontrolled symptoms or poor clinical outcomes only if human resources are a limitation” was added in R2. The consensus agreement dropped slightly from 97.7% in R1 to 90.6% in R2. The study team adopted the statement in R2 by considering the panellists’ comments and patient census (Table 3).

No significant differences were found between rounds, as shown in the Mann–Whitney U test (*p* = 0.317–1.000) (Table 3). The full pharmacist-led educational material is available in Additional file 1: Appendix II: Pharmacist-led education material.

### Educational video evaluation

The third study phase recruited 79 participants diagnosed with AR, where the median age of the respondents was 47.00 (IQR: 33). The majority were female (60.8%), had secondary school education (50.6%), and had Malay ethnicity (54.4%).

Overall, the education material was “understandable” and “actionable”; the material secured at least 70% for each domain. Of 11 items under the “understandability” domain, 6 scored 100%, 3 scored 98.7%, and 1 scored 97.5% and 89.9%, respectively. Although all items achieved the minimum score of 70%, the least agreement rated was on ‘the material allows the user to hear the words clearly (e.g., not too fast, not garbled)’. Meanwhile, the “actionability” domain secured scores of 100% for all items (Table 4).

**Table 4:** The items of “understandability” and “actionability” of the patient education material

Items		*n* = 79 *n* (%)
Understandability		
Content	The material makes its purpose completely evident	78 (98.7)
Word choice and style	The material uses common, everyday language	79 (100)
	Medical terms are used only to familiarize audience with the terms. When used, medical terms are defined	77 (97.5)
Organization	The material uses the active voice	79 (100)
	The material breaks or “chunks” information into short sections	79 (100)
	The material’s sections have informative headers	78 (98.7)
	The material presents information in a logical sequence	79 (100)
	The material provides a summary	78 (98.7)
Layout and design	Text on the screen is easy to read	79 (100)
	The material allows the user to hear the words clearly (e.g., not too fast, not garbled)	71 (89.9)
Use of visual aids	The material uses illustrations and photographs that are clear and uncluttered	79 (100)
Actionability		
The material clearly identifies at least one action the user can take		79 (100)
The material addresses the user directly when describing actions		79 (100)
The material breaks down any action into manageable, explicit steps		79 (100)
The material explains how to use the charts, graphs, tables, or diagrams to take actions		79 (100)

## Discussion

This study developed a pharmacist-led education model using a multi-phase approach. One of the approaches used, the Delphi technique, was convenient, time- and cost-efficient for panellists from diverse backgrounds [42]. The Delphi technique emerged and retained important competencies after reflection while restructuring less clearly defined ones [43, 44]. This study assessed group stability, internal agreement, and inter-rater reliability, contrasting previous studies that reported consensus agreement in percentage [31] or median score [45].

The study adopted a two-round modified Delphi process, with structured items and a 9-point Likert scale in the first round. This approach simplifies consensus-building and is suitable for generating consensus on critically important topics for patients [25]. The quantitative approach was adapted based on the availability of basic information from the pharmacist-led educational model [23, 24]. This method can be applied when the information needed to be delivered to patients is broad that panellists are needed to reach a consensus [46].

All items met the standard for consensus agreements among panellists in the final round. Nonetheless, two items under “allergen avoidance” did not achieve consensus due to impractical action and unclear instructions in R1. The item indicating “Wearing goggles” was incomplete and unclear, so it was clarified as “Wear wrap-around sunglasses when being outside.” The item “Ensuring the door and window are kept closed” was unrealistic in real life. The panellists suggested closing windows and doors when outdoor air quality was poor and providing examples of poor air quality. Consensus agreements were achieved for these items in R2, but slight instability was noticed for these two items. The application of VCV suggested an internal agreement was reached for these items in R2. Additionally, an improvement in the proportion of panellists rating items with scores of “7 to 9” was observed, indicating that the items were critically important to be included in the model.

The second part of the model focuses on pharmacist counselling scopes and algorithms. In R1, a satisfactory consensus agreement was achieved, and all domains remained stable. However, some suggestions for statement improvement were adapted for R2, resulting in higher consensus agreement. For example, the panellist suggested recruiting all patients into the pharmacist-led education model instead of only adult patients with moderate-to-severe AR. This was based on the substantial patient load at outpatient pharmacies, which refilled 44.9 million prescriptions in 2021 in the public healthcare setting [47]. Improvements were observed in R2, with an increase in “consensus agreement” from 88.1% to 90.6% and a reduction in the width of the IQR from 7.25–9.00 to 8.00–9.00.

The stepwise pharmacological management approach and disease classification adopted from the ARIA guidelines raised concerns among panellists, who assumed a shift from physicians to pharmacists in patient treatment decisions. The statement in R1 was restructured in accordance to the level of agreement of the panellist to include this information in the pharmacist counselling. The panellists might misunderstand that the pharmacists would change the regimes by following the stepwise pharmacological treatment according to the disease severity. The R2 rating explained that the inclusion was only for the knowledge of practising pharmacists, with the concern that most pharmacists had inadequate awareness of ARIA guidelines (40.4%) [48]. The revised version assuring the panellist that there is no shift of the role of physicians to pharmacists in the treatment decision.

This study tested the understanding of patient education material in video format on patients at the final phase, enhancing its robustness. Educational videos were the most effective medium for improving knowledge in an easy-to-understand way [49]. The study converted the patient education material from text to audio-visual format to facilitate patient understanding [49–51]. A local study found that providing patient education via video had the highest impact on nasal spray usage [52]. However, the video mainly focused on nasal spray administration without addressing other aspects of AR management.

Patients with adequate information about their diseases and management strategies are more proactive in medication intake and controlling symptoms [7]. However, 31% of YouTube’s videos on AR were found unreliable and may negatively impact patient outcomes [53]. Access to legitimate sources is also a concern due to differences in educational background and patient health literacy levels. Patient education information varies slightly among online references [7, 54–59]. Hence, having the material validated by an expert panel to ensure its validity and usability is crucial. Testing the material on patients further confirms its credibility.

This study developed a pharmacist-led education model to provide comprehensive information on AR and counselling. In contrast, the previous studies focused on educating the pharmacist and their assistant through intensive training and workshops [16, 28, 60]. Our study model includes patient education, healthcare professional support, and patient selection criteria for pharmacists to support AR patient management in public health services settings. The model also includes specific parameters like symptom control, quality of life, and nasal spray administration technique assessment. This approach may give greater patients self-assurance in their ability to cope with AR and achieve desired symptom control levels [18].

The Patient Education Materials Assessment Tool (PEMAT-A/V) was utilized to assess patients’ understandability and actionability after watching the video. Unlike other tools, PEMAT-A/V evaluates content dimensions, word choice, style, organization, layout, design, and visual aids [39]. Suitability Assessment of Materials (SAM) was also used to test patient education materials, but focusing more on instruction and actionability [61]. The Educational Content Validation Instrument in Health that tests educational content in video was considered for adaptation, but its usability could be limited due to its focus on written content without considering images, graphics, or drawings [62].

### Limitations

The choice to involve local panellists may restrict the applicability of this model on a global scale. Paradoxically, this limitation can be viewed as a strength of the study, as local panellists possess an in-depth understanding of the specific nuances and practices within the local context, thereby enhancing the model's relevance and effectiveness within that specific region [31]. The use of consecutive sampling in phase 3 lacked the nature of probability sampling. However, the inclusion of all accessible participants would be considered most reflecting the common scenario in a clinical setting, and it is a better choice of non-probability sampling as compared to convenience sampling.

## Conclusion

The pharmacist-led education model in managing patients with AR achieved consensus agreement among the expert panels, with good stability and within-range VCV between rounds. Two items indicated lesser stability remained in the model, with most of the panels deemed that these items were critically important and further supported by acceptable VCV. Therefore, this model is a valid tool for healthcare providers, especially among pharmacists conducting educational interventions in patients with AR. Most importantly, a high level of understandability and actionability of the educational material among the targeted population, i.e. patients with AR, would promote the adaption of this model into daily clinical practice and potentially serve as a standard guide for healthcare providers in patient education.

### Supplementary Information

**Additional file 1: Appendix I.** Rating of panellist in R1 survey.** Appendix ll.** Pharmacist-led education material.

## Data Availability

The data belonged to Clinical Research Centre and the Perak State Health Department under the Malaysian Ministry of Health. Hence, it cannot be shared publicly. However, with a reasonable request, should any party require the data, they can send their request to the corresponding author with permission from the Director General of Health prior to being shared with any party.
