# Supplementary material for: Development and validation of a pharmacist-led education model in allergic rhinitis management: a multi-phase study
Source: J Pharm Policy Pract. 2023 Oct 4;16:116. doi: 10.1186/s40545-023-00625-1 (PMC10548631; doi:10.1186/s40545-023-00625-1)
Supplement: Supplementary file 1 — Additional file 1: Appendix I. Rating of panellist in R1 survey. Appendix ll. Pharmacist-led education material. [file 40545_2023_625_MOESM1_ESM.docx]

# Appendix I: Rating of panellist in R1 survey

| Domains | No. of items | Mean | SD | Min | Max | Median score | Q1 | Q3 | Critically important | Important but not critical | Not important |
| --- | --- | --- | --- | --- | --- | --- | --- | --- | --- | --- | --- |
| **Patient educational material** |  |  |  |  |  |  |  |  |  |  |  |
| 1. Knowledge of the disease | 4 | 8.36 | 1.06 | 3 | 9 | 9.00 | 8.00 | 9.00 | 95.9% | 2.9% | 1.2% |
| 1. Symptoms assessment | 5 | 8.16 | 1.07 | 4 | 9 | 8.40 | 7.60 | 9.00 | 92.1% | 7.9% | 0.0% |
| 1. Diagnosis | 5 | 7.90 | 1.39 | 3 | 9 | 8.60 | 7.50 | 9.00 | 86.0% | 11.2% | 2.7% |
| 1. A) Allergen identification and avoidance | 17 | 7.80 | 1.44 | 2 | 9 | 8.24 | 7.06 | 9.00 | 75.4% | 22.6% | 2.0% |
| B) Two items of allergen identification and avoidance | 2 | 6.79 | 2.18 | 1 | 9 | 7.50 | 5.00 | 9.00 | 64.0% | 31.4% | 4.7% |
| 1. Nasal corticosteroid | 15 | 7.98 | 1.41 | 1 | 9 | 8.53 | 7.50 | 9.00 | 88.4% | 9.4% | 2.2% |
| 1. Technique of priming | 5 | 8.48 | 0.87 | 3 | 9 | 9.00 | 8.00 | 9.00 | 92.1% | 6.5% | 1.4% |
| 1. Before administration | 8 | 8.33 | 1.13 | 1 | 9 | 8.94 | 8.00 | 9.00 | 91.9% | 7.6% | 0.6% |
| 1. During administration | 4 | 8.35 | 0.93 | 5 | 9 | 9.00 | 8.00 | 9.00 | 92.6% | 7.4% | 0.0% |
| 1. After administration | 2 | 8.38 | 1.00 | 5 | 9 | 9.00 | 8.00 | 9.00 | 91.9% | 8.2% | 0.0% |
| 1. Tips of usage | 5 | 8.21 | 1.26 | 3 | 9 | 9.00 | 8.00 | 9.00 | 88.8% | 9.3% | 1.9% |
| 1. Cleaning | 6 | 8.11 | 1.32 | 2 | 9 | 8.83 | 7.50 | 9.00 | 85.8% | 13.4% | 0.8% |
| 1. Antihistamine | 10 | 8.17 | 1.17 | 1 | 9 | 8.90 | 7.45 | 9.00 | 87.2% | 12.1% | 0.7% |
| 1. Decongestant | 19 | 8.14 | 1.26 | 1 | 9 | 8.79 | 7.58 | 9.00 | 88.9% | 9.7% | 1.5% |
| 1. Nasal douche (Saline) | 13 | 8.17 | 1.09 | 2 | 9 | 8.77 | 7.46 | 9.00 | 90.7% | 8.8% | 0.5% |
| 1. What to do when symptom flare | 6 | 8.17 | 1.16 | 2 | 9 | 8.83 | 7.33 | 9.00 | 90.7% | 8.1% | 1.2% |
| 1. Consequences of non-adherence | 4 | 8.21 | 1.05 | 5 | 9 | 8.75 | 7.63 | 9.00 | 91.3% | 8.7% | 0.0% |
| **Pharmacist counselling scopes and algorithm** |  |  |  |  |  |  |  |  |  |  |  |
| 1. Patient selection criteria | 1 | 7.88 | 1.37 | 4 | 9 | 8.00 | 7.25 | 9.00 | 88.1% | 11.9% | 0.0% |
| 1. Symptoms control assessment | 1 | 8.00 | 1.29 | 3 | 9 | 8.00 | 7.50 | 9.00 | 90.5% | 7.1% | 2.4% |
| 1. Patients’ quality of life | 1 | 8.23 | 1.01 | 6 | 9 | 9.00 | 8.00 | 9.00 | 89.7% | 10.3% | 0.0% |
| 1. Setting goal of treatment | 1 | 8.21 | 0.84 | 6 | 9 | 8.00 | 8.00 | 9.00 | 95.2% | 4.8% | 0.0% |
| 1. New user to nasal spray | 2 | 8.57 | 0.67 | 7 | 9 | 9.00 | 8.00 | 9.00 | 100.0% | 0.0% | 0.0% |
| 1. Existing nasal spray users | 2 | 8.56 | 0.65 | 6 | 9 | 9.00 | 8.00 | 9.00 | 98.8% | 2.4% | 0.0% |
| 1. Both new and existing user | 5 | 8.38 | 0.91 | 4 | 9 | 9.00 | 8.00 | 9.00 | 96.7% | 3.3% | 0.0% |
| 1. Teach patients the alert sign | 3 | 8.37 | 0.93 | 4 | 9 | 8.83 | 7.67 | 9.00 | 95.2% | 4.7% | 0.0% |
| 1. Follow-up pharmaceutical care | 1 | 8.26 | 1.17 | 4 | 9 | 9.00 | 8.00 | 9.00 | 92.9% | 7.1% | 0.0% |
| 1. Patient discharge | 1 | 8.24 | 1.16 | 4 | 9 | 9.00 | 8.00 | 9.00 | 95.2% | 4.8% | 0.0% |
| 1. Algorithm of pharmaceutical care | 1 | 8.13 | 1.03 | 5 | 9 | 8.00 | 8.00 | 9.00 | 92.3% | 7.7% | 0.0% |
| 1. Addressing concern of treatment | 6 | 8.37 | 0.93 | 3 | 9 | 8.92 | 8.00 | 9.00 | 98.0% | 0.8% | 1.2% |
| 1. Medication adherence | 4 | 8.53 | 0.69 | 7 | 9 | 9.00 | 8.00 | 9.00 | 100.0% | 0.0% | 0.0% |
| 1. Stepwise treatment approach | 1 | 8.00 | 1.55 | 2 | 9 | 9.00 | 8.00 | 9.00 | 86.5% | 10.8% | 2.7% |
| 1. Pharmacotherapy agents | 13 | 8.28 | 0.99 | 2 | 9 | 8.88 | 7.92 | 9.00 | 87.1% | 12.7% | 0.2% |

The number of items represents the total number of items attributable to a specific domain.

The median score represents the midpoint of the score distribution for a particular domain.

Q1 and Q3 indicated the first and the third quartile of the median scores

A consensus is reached when at least 70% of the panellists score in the same range (either critically important, important but not critical or not important)

# Appendix Il: Pharmacist-led education material

| *Part A: Content of patient educational material on the management of allergic rhinitis* |
| --- |
| Disclaimer:  This patient education material does not replace medical advice from your health care provider. Talk to your health care provider if you have any questions about condition or your treatment. |
| 1. Knowledge about allergic rhinitis |
| - 1. *Background*   Allergic rhinitis is an inflammatory condition in the nose that occurs when an individual expose to an allergen. |
| - 1. *Causes*   It is caused by overreaction of the immune system to an allergen such as pollen, fungus, animal dander, smokes (tobacco smoke, diesel fumes) or certain foods (seafood, seasonings etc.). |
| - 1. *Risk factor*   People who have asthma, eczema, or their family member with these conditions are more likely to get allergic rhinitis. |
| - 1. *Disease nature*   It can begin at any age. Most people have the symptoms in their childhood or in teens, and it is usually long-standing condition. |
| 1. Symptoms of Allergic Rhinitis |
| - 1. Symptoms usually appear within minutes of exposure to an allergen. |
| - 1. The symptoms vary from person to person: - Nose: runny nose, blocked nose, sneezing, itching |
| - Throat and ears: sore throat, congestion of the ears, itching of the throat or ears. |
| - Eyes: itchy, red eyes, grittiness, watery eyes, swelling and dark circles beneath the eyes. |
| - Sleep: mouth breathing, frequent awakening, daytime fatigue. |
| - Activity: trouble doing normal activities (e.g., work) |
| 1. Diagnosis |
| The doctor will conduct :-   - Evaluation of medical, social and family history. |
| - Physical examination. |
| - Rhinoscopy or nasal endoscopy. |
| - Skin prick test or serum testing. |
| - **Each of these tests are based on the doctor’s judgement/based on the severity of allergic rhinitis |
| 1. Allergen avoidance strategy |
| - 1. *Identifying allergen* - Keeping a diary to identify allergens: where and what you were doing before your symptoms started. |
| - Examples of allergen - Indoor: animal dander, fungal spores. - Outdoor: pollen, diesel fumes. |
| - 1. *Reduction of indoor allergen*   The methods to reduce allergens in the house are as follows:   - Allergen-impermeable bedding cover. |
| - Wash bedding at least once every two weeks. |
| - Clean the floor surface daily with water. |
| - Avoid soft furnishing and furry stuff at home such as curtain, cushions, soft toy, and carpet. |
| - Keep decorations, books, and clothes in cabinets. |
| - Significantly reduce symptoms in 6 months once the pets are moved to outdoor. |
| - If pets have to be kept indoors, keep them away from bedding and the sofa. |
| - Washing pets’ bedding, soft furnishing they have been on regularly |
| - 1. *Removal of outdoor allergen* - Shower and change clothes after being outside |
| - To stay indoors when outdoor air quality is poor (e.g., haze, fumes) |
| - Wear wrap-around sunglasses (e.g., goggles) when being outside. |
| - Keep doors and windows closed when outdoor air quality is poor. |
| - 1. *Face mask* - Standard 3 ply surgical masks filter such as fungal spores, pollen |
| - Wear a face mask whenever there is a risk of being exposed to indoor or outdoor allergen. |
| - For example: when cleaning the house or going to area with poor air quality or a pollen-rich area. |
| - 1. *Air purifier with HEPA filter* - It reduces allergen level and may reduce symptom of allergic rhinitis. |
| - Nevertheless, wet cleaning floor and surfaces are required. |
| - Note: Effective allergen reduction can be achieved by practicing a combination of the above methods. |
| 1. Nasal steroid spray |
| - 1. *General information* - It contains steroids to reduce nasal inflammation. |
| - It helps you to stay free of symptoms. |
| - It is a first-line therapy to treat the symptom sneezing, itching, runny and blocked nose. |
| - It has to be used on a regular basis by following the doctor’s instructions, even on days you do not experience any symptoms. |
| - 1. *Effect* of medication - The effectiveness of the medicine can be detected within several hours to several days. |
| - An antihistamine will be taken together to increase the effectiveness of steroids in symptom reduction. |
| - The optimal effect will be seen in 2 weeks. |
| - 1. *Expectations from treatment* - A complete cure of this problem may not be possible, but it can be used to control the symptoms. |
| - However, the doctor will plan for a step-down treatment of nasal steroid spray when the symptoms are improving. |
| - 1. *Concern of prolonged use* - Continuous use of intranasal steroid spray will not cause loss of effectiveness over time. |
| - If you think the nasal steroid spray isn't working adequately, see your doctor. |
| - 1. *Side effect* - Headache - A mildly unpleasant smell and taste - Nose or throat dryness   Nota: Correct use of technique can keep these side effects to a minimum. |
| - Side effects are often limited to the start of treatment. If you continue to experience the side effects, please consult your doctor. |
| - Side effects such as nasal dryness, can be improved with the use of moisturizing creams or ointments |
| - Absorption of the drug to other parts of the body is very minimal (approximately 0.5% only). Therefore, there are no significant long-term side effects because it is only used locally. |
| - 1. *How to prime the nasal steroid spray*  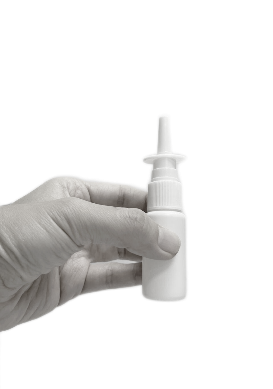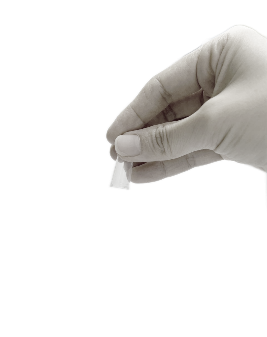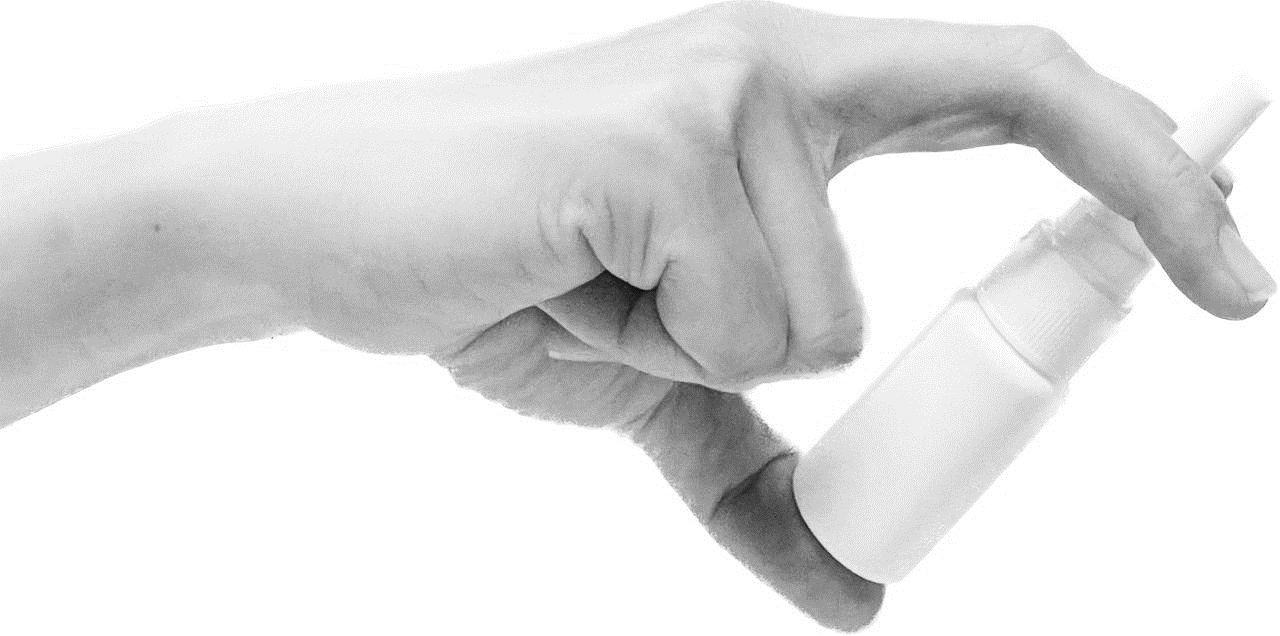 *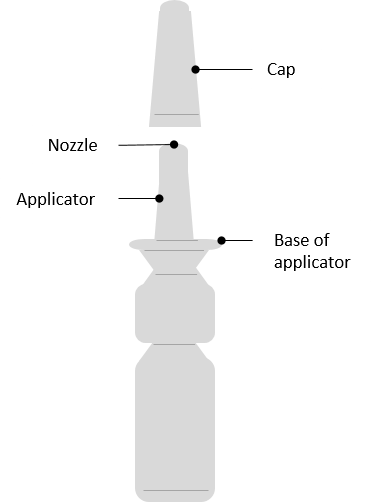*  *Figures adapted from Malaysian Official Portal MyHealth, available at: http://www. myhealth.gov.my/en/how-to-use-nasal-spray/*  Picture A  Picture B   1. Shake the bottle and remove the cap (picture A). |
|  |
| 1. Hold the bottle vertically. Place your thumb underneath the bottle and place your index and middle fingers at the base of applicator (picture B) |
| 1. Point the nozzle away from you. 2. Press down on the base of the nasal applicator to release the spray. 3. Press 3 to 5 times until a uniform spray appears (the number of presses depends on the type of nasal spray device). This process is called priming (Picture B). |
| Note: Priming is only done once for every nasal spray. However, if the nasal spray has not been used for more than 1-2 weeks, the priming step has to be repeated. |
| - 1. *General Instructions for use of nasal steroid spray (Patients should follow the product leaflet for the specific product instructions).*   Before use   1. Wash your hands |
| 1. Gently blow your nose to clear the mucous |
| 1. Shake the bottle and remove the cap. |
| 1. Hold your head upright |
| 1. Use the RIGHT hand for the LEFT nostril. |
| 1. Hold the nasal spray upright, thumb placed underneath the bottle, and index and middle fingers placed on the base of the nasal applicator. |
| 1. Carefully insert the applicator tip into the other nostril |
| 1. Point the nozzle of the nasal spray away from the centre of the nose and towards the outer corner of the eye on the same side of the nose. |
| During   1. Press the pump 1 time only, at the same time, slowly inhale through the nose. |
| 1. Remove the nozzle from the nostril and exhale slowly through the mouth    - Do not tilt your head backwards to prevent backflow into your throat. |
| - - If the medicine drips into your throat, you will feel a slightly bitter sensation in your mouth. This is normal. Do not need to repeat the dose. You need to be more cautious when applying the next spray. |
| 1. If instructed to take 2 pumps in each nostril, wait for 10 - 15 seconds before taking the second pump in the same nostril. |
| 1. Switch hands and repeat steps 6-11 for the other nasal cavity. |
| After use   1. Wipe the applicator tip with a clean tissue or cloth and put on the plastic cap. |
| 1. Try not to sneeze or blow your nose immediately after using the nasal spray. |
| - 1. Tips *While* Using The Nasal Spray - The effects of nasal steroid sprays work best when medication remains in the nose. |
| - If the nasal spray is used correctly, the medicine will not drip from your nose nor will it drip into your oral cavity. |
| - If you experience any nosebleed, stop using the nasal spray for a few days and use nasal saline irrigation. - If the bleeding continues, please seek medical attention. |
| - Keep the nasal spray away from direct sunlight and children. |
| - 1. *How to* clean *nasal steroid spray*   You will need to clean it regularly.   - Remove the cap and gently pull off the nozzle. |
| - Wash the nozzle and cap in warm water and then rinse under a running tap. |
| - DO NOT try to unblock the nasal applicator by inserting a pin or other sharp object as this will damage the applicator and cause you not to get the right dose of medicine. |
| - Allow the cap and nozzle to dry. |
| - Push the nozzle back onto the bottle and cap the nozzle. |
| - Please remember to bring along your empty bottle to exchange for a new bottle in the government hospital or clinic |
| 1. Oral antihistamine |
| - 1. *Expectation of treatment effects* - The symptoms may be reduced as soon as 15 minutes to 1 hour. |
| - Only taking oral antihistamine may not adequately control the symptoms. You will need to use nasal steroid spray to adequately control the symptoms. |
| - 1. *Side effects* - You may have dry mouth. |
| - DO NOT drive or operate machinery when you are taking this medicine. - Although the doctor will prescribe you non-drowsy medicine, some people may feel drowsy with these products. Please check with the doctor or pharmacist. |
| - Before taking antihistamine, please inform the doctor or pharmacists if you have other medical condition (e.g, glaucoma, prostate or thyroid problems) |
| 1. Decongestant *(medicine relieves nasal congestion)* |
| - 1. *General information* - It reduces blocked nose. |
| - 1. *Oral Decongestant* - Oral decongestant is available in combination with oral antihistamines. |
| - It should be taken orally as instructed by the doctor. |
| - They are best taken in the morning as some people may experience nervousness, or difficult to sleep after taking the medications |
| - Oral decongestants are not suitable for people with high blood pressure, certain heart disease conditions, glaucoma, or thyroid problems. If you have any of these conditions, please consult a doctor or pharmacist before using this medication. |
| 1. Decongestant Hidung *(Ubat melegakan hidung tersumbat)* |
| - 1. *Decongestant hidung* - It provides faster relief of blocked nose compare to oral decongestant. |
| - Some people may need to use a decongestant to reduce blocked nose in order to allow the nasal steroid spray to reach more areas within the nose. |
| Instruction of use   1. Hold the head upright. |
| 1. Place the spray nozzle into the nostril without completely occluding the nostril. |
| 1. Squeeze bottle firmly. |
| 1. Do not tilt head backward while spraying. |
| 1. Wipe nozzle clean after use. |
| 1. Secure cap after use. |
| Dosage   - Take 2 or 3 sprays into each nostril twice daily, morning and evening. |
| - Do not use the nasal spray more than 2 times in 24 hours. |
| - It is for short term use. Please follow the doctor's instructions. |
| - Excessive use may cause blocked nose again. |
| Side effects   - Mild side effect include stinging, sneezing or increased discharge of the nose. |
| 1. Nasal saline irrigation |
| - 1. *General information* - A saline solution that does not contain any medication. |
| - It helps to clear allergens from the nose. |
| - It can be used to clear nasal discharge first before using a nasal steroid spray. This would allow better medication absorptions. |
| - It is beneficial in treating runny noses and may be used alone or as an add-on therapy. |
| - 1. *Dosage* - It can be used when needed. |
| - Note: to follow each product instruction of use or ask the pharmacist for more information. |
| - 1. *General instruction* - Note: Always use water that has been disinfected or boiled and allowed to cool to mix the powder for nasal irrigation to reduce the risk of infection. |
| 1. Stand in front of a basin |
| 1. Tilt your head forward, pump the solution into each nostril |
| 1. The solution may flow into one nostril and out the other. |
| 1. Avoid breathing through the nose while doing irrigation, as this can introduce water into the ear canal and lung, potentially leading to infection. |
| - 1. *Side effects* - It reduces symptom without side effect. |
| 1. What to do when symptom flare? |
| If you have symptoms flaring up or becoming more severe such as frequent sneezing and runny hose, try the following method: -   - Take an oral antihistamine immediately to relieve the symptoms. |
| - Although symptoms can be reduced within 30 minutes, you must concurrently administer nasal steroids spray as instructed by the doctor. |
| - Note: taking oral anti-histamines alone does not completely control the symptoms, it only provides temporary relief. Nasal steroid sprays are the main treatment for nasal allergies. |
| - Try identifying and avoiding allergens |
| - If symptoms is not improving in 24 hours, please visit your nearest clinic. |
| - Warning signs: If you feel shortness of breath or chest tightness, you need to go to the emergency at the hospital. |
| 1. Akibat alahan hidung yang tidak dikawal dengan baik |
| - This could lead to sinusitis and causes growth inside the nose or fluid in the middle ear. |
| - It could cause excessive breathing through the mouth. Thereby causing sleep disturbance, snoring, or dental. |
| - It may worsen asthma control if someone who has co-existing of asthma and allergic rhinitis. |
| - It could compromise performance in work or school. |

| ***Part B***  ***Scope of pharmacist counselling for adult patients***  Pharmacist Counselling Scopes include:   - Counselling guide. - General information about allergic rhinitis management. |
| --- |
| 1. Patient selection criteria  - All adult patients with allergic rhinitis (subject to feasibility, e.g., human resource limitations of the healthcare institutions). - If feasibility is an issue, consider selecting adult patients suffering from moderate to severe allergic rhinitis. |
| 1. Symptoms control assessment and monitoring   (Retrieved from the patient's note or real-time assessment).   - Patient disease severity assessment in terms of frequency and severity using the Allergic Rhinitis and its Impact on Asthma (ARIA) guideline classification.   *Frequency:*  *Intermittent: < 4 days per week or < 4 weeks at a time; Persistent: ≥ 4 days per week, and ≥ 4 weeks at a time.*  *Severity:*  *Mild: Normal sleep, daily activities, work/school and no troublesome symptoms;*  *Moderate to severe: One or more of the symptoms including abnormal sleep, impairment of daily activities, sport, leisure, and problem at work or school, troublesome symptoms.* |
| 1. Assessing patients’ quality of life   (Retrieved from the patient's note or real-time assessment). |
| 1. Setting goal of treatment:  - Aim to maintain symptom free or at least mild level of severity. |
| 1. New user to corticosteroid nasal spray  - Demonstrate the corticosteroid nasal spray technique in:-   - 1. Priming     2. Administration     3. Cleaning   (Please refer to the types of intranasal corticosteroid product in the pharmacotherapy agent page). |
| - Explain the expected effects of nasal steroid spray. |
| 1. Existing nasal steroid spray users  - Evaluate patient’s corticosteroid nasal spray administration technique and correct it when necessary. - Consider evaluating patients with uncontrolled symptoms or poor clinical outcomes only if human resources are a limitation. |
| - Assess patients’ nasal spray adherence. |
| 1. Both new and existing user  - Assess and address patients’ concerns with the treatments. |
| - Note: To discuss with the medical doctor in-charge if uncertain about the queries or patients are found to be having problems with medication adherence. |
| - Explain the importance of adherence and the consequences of non-adherence. |
| - Emphasize the importance of allergen identification and avoidance. |
| - Encourage patients to access the patient education materials from time to time. |
| 1. Teach patients the alert sign.  - Some patient have AR co-existing with asthma. Please assess if patients have both of these diseases. |
| - Emphasise the importance of adherence to asthma therapy (inhaler), if patients have AR co-existing with asthma. Non-adherence to the inhaler would worsen allergic rhinitis, and vice versa. |
| - Note: Patients with asthma exacerbations will have difficulty breathing and/or chest tightness; advise the patient to go to the emergency room right away. |
| 1. Follow-up care by pharmacists (when referral by the physician).  - Perform step 4 to 6. |
| 1. Patient discharge from the pharmacists’ follow-up  - Patients can be discharged from pharmacists’ follow-up based on the physician’s assessment. |


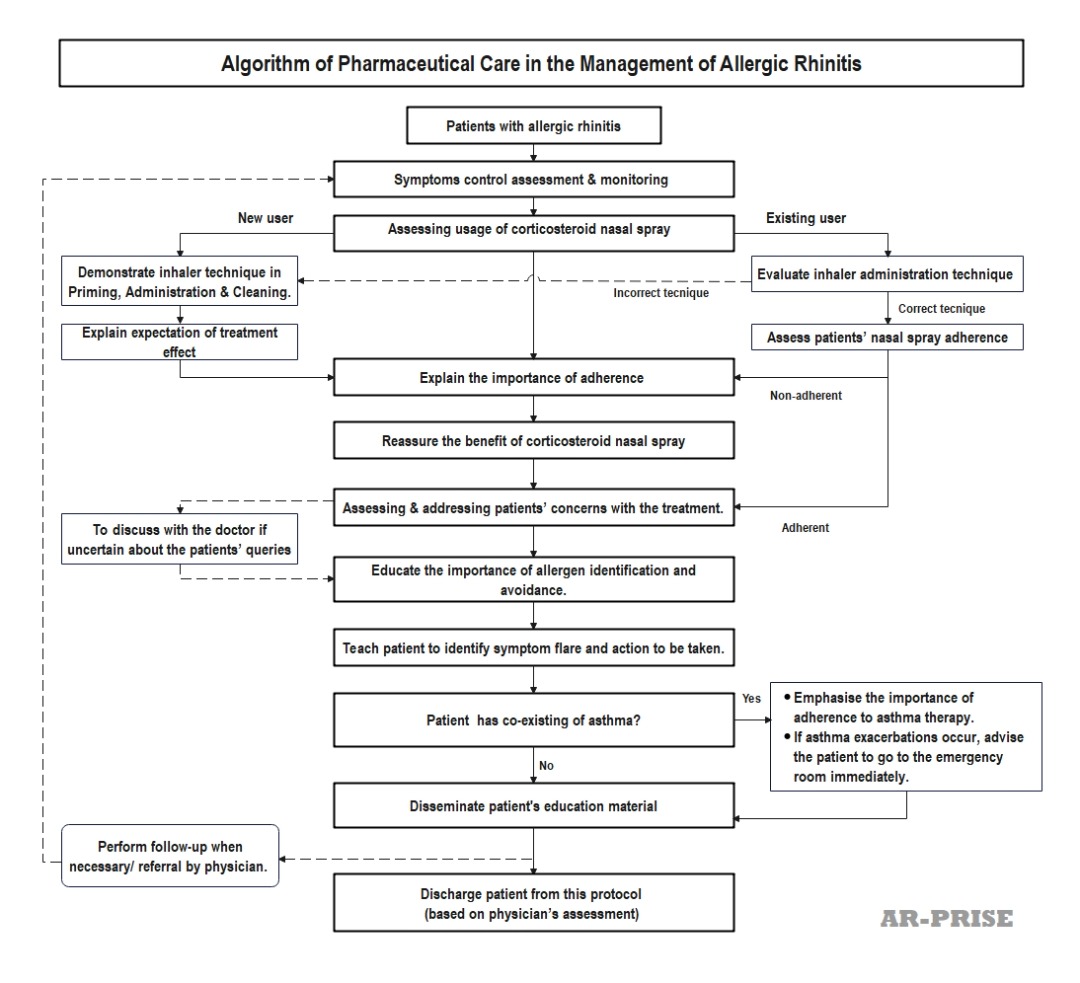


**General knowledge for pharmacists’ understanding**

The inclusion of a stepwise treatment approach and disease classification (below) is for the general knowledge of pharmacists. (Pharmacists are NOT involved in the patient's stepwise treatment approach).

Source: adapted from the ARIA guideline; 2019 [19]

| **Frequency of symptoms**  **The possible classifications:**   1. Intermittent mild 2. Intermittent moderate to severe 3. Persistent mild 4. Persistent moderate to severe | **Intermittent symptoms** |  | **Persistent symptoms** |
| --- | --- | --- | --- |
|  | - <4 days per week or - <4 weeks |  | - >4 days per week and - >4 weeks |
|  |  |  |  |
| **Disease severity** | **Mild** |  | **Moderate-to-severe** |
|  | All of the following are present |  | One or more of the following are present |
|  | - Normal sleep - Normal daily activities, leisure and/or sport; - Normal school or work function - No troublesome symptoms |  | - sleep disturbance - impairment of daily activities, leisure and/or sport - impairment of school or work; - troublesome symptoms |

**Stepwise Treatment Approach**

(Adults & Adolescents)

**Confirm diagnosis of Allergic Rhinitis**

Assess for **asthma** (especially among patients with severe and/or persistent rhinitis

**Intermittent symptoms**

**Persistent symptoms**

**Moderate-severe**

Not in preferred order

- Oral H1 Blocker
- Or intranasal H1 Blocker and/or decongestant
- Or INCS
- Or LTRA*
- Or Chromone

**Mild**

**Moderate-severe**

**Mild**

Not in preferred order

- Oral H1 Blocker
- Or intranasal H1 blocker and/or decongestant
- Or LTRA*

Review the patients in 2-4 weeks in the case of persistent rhinitis

If symptom NOT improved: step up treatment.

If symptom improved: continue treatment for one month

In preferred order

- INCS
- H1 Blocker or LTRA*

Review the patient in 2-4 weeks

Symptoms improved

Symptoms NOT improved

Step down and continue treatment for more than one month

Add or increase INCS doses

Rhinorrhea

add ipratropium

Blockage

add decongestant or short-term oral corticosteroid

Failure: Refer to specialist

Review

- Diagnosis
- Compliance

Query

- Infections
- Other causes

**Allergens Avoidance**

If conjunctivitis, add

- Oral H1 blocker
- Or intraocular H1 blocker
- Or intraocular chromone
- Or saline

Consider immunotherapy

Source: adapted from the recommendation of ARIA guideline; 2012 [62]

LTRA: Leukotriene receptor antagonist, INCS: Intranasal corticosteroid

**General knowledge for pharmacists’ understanding**

Pharmacotherapy agents for allergic rhinitis patients’ management

The pharmacotherapy agents, indications and dosages are adapted from Ministry of Health Medicines Formulary.

*(Source: Ministry of Health Medicines Formulary – Bil.1/2022)*

*The medication listed is the general dosage. These dosages will be revised depending on severity or when newer dosages become available.* (This is general information for the pharmacist, not for patient counselling).

| Generic Drug Name | Indications | Dosage |
| --- | --- | --- |
| Antihistamine | | |
| Cetirizine HCl  10 mg Tablet | Perennial rhinitis, allergic rhinitis. | 10 mg daily or 5 mg twice daily. |
| Desloratadine  5 mg Tablet | Allergic rhinitis. | 5mg once a day regardless of mealtime. |
| Dexchlorpheniramine Maleate  2 mg Tablet | Symptomatic treatment of allergic rhinitis. | 2 mg 3 times daily. |
| Levocetirizine Dihydrochloride  5 mg Tablet | Symptomatic treatment of allergic rhinitis (including persistent allergic rhinitis) | 5 mg orally once daily (Swallow whole, do not chew / crush). |
| Loratadine  10 mg Tablet | Allergic rhinitis | 10 mg once daily. |
| Generic Drug Name | Indications | Dosage |
| Combination preparation | | |
| Loratadine 5 mg and Pseudoephedrine Sulphate 120 mg  Tablet | For treatment of allergic rhinitis. May be more effective than either product alone but side effects are combined. | 1 tablet twice daily |
| Azelastine Hydrochloride 137mcg and  Fluticasone Propionate 50mcg  Nasal Spray | Symptomatic treatment of moderate to severe allergic rhinitis and rhino-conjunctivitis where use of a combination (intranasal antihistamine and glucocorticoid) is appropriate.  As a second line treatment: only for those whose symptoms remain uncontrolled on oral antihistamine or intranasal corticosteroids (INS) monotherapy, or on a combination of oral antihistamine plus INS. | One actuation in each nostril twice daily |
| Intranasal corticosteroid (INS) | | |
| Budesonide  64mcg Nasal Spray | Seasonal and perennial allergic rhinitis. | 2 sprays into each nostril once daily in the morning or 1 spray into each nostril twice daily. |
| Fluticasone propionate  27.5 mcg/dose nasal spray | Treatment of nasal symptoms (rhinorrhea, nasal congestion, nasal itching and sneezing) and ocular symptoms (itching/ burning, tearing/ watering, and eye redness) of allergic rhinitis. | 1-2 sprays (27.5 mcg/spray) in each nostril once daily. |
| Mometasone furoate 50 mcg/dose aqueous nasal spray | Allergic rhinitis | 100 mcg/day (2 sprays) to each nostril once daily.  Maximum 200 mcg (4 sprays) once daily. Reduce to 50 mcg (1 spray) once daily when control achieved. |
| *Leukotriene receptor antagonists (LTRAs)* | | |
| Montelukast Sodium 10 mg Tablet | Chronic treatment of asthma and relief of symptoms of allergic rhinitis for adolescent (≥ 15 years) and adults. | 10 mg daily at bedtime. |
| *Adjunct Therapy* | | |
| Alkaline Nasal Douche | To remove nasal plug | To be diluted with an equal volume of warm water before use |
| *Ocular preparation* | | |
| Sodium Cromoglycate 2% Eye Drops | Prevention and treatment of allergic conjunctivitis including allergic conjunctivitis and vernal keratoconjunctivitis | 1 or 2 drops 4 times daily |
